# Community Perceptions and Attitudes Toward Vaccination in Madagascar

**DOI:** 10.3390/vaccines14020191

**Published:** 2026-02-19

**Authors:** Maharisoa Ralambosoa, Amandine Oleffe, Vatsiharizandry Mandrosovololona, Zo Patricia Rasolomanana, Lethicia Lydia Yasmine, Paubert Tsivahiny, Mamy Andrianirina Rakotondratsara, Laurent Musango

**Affiliations:** 1 WHO Madagascar, United Nations Common House, Galaxy Zone Andraharo, P.O. Box 362, Antananarivo 101, Madagascar; vmandrosovololona@who.int (V.M.); rasolomananap@who.int (Z.P.R.); rakotondratsaram@who.int (M.A.R.); musangol@who.int (L.M.); 2 Research Centre on Health Policy and Systems—Global Health, School of Public Health, Université Libre de Bruxelles, 1050 Brussels, Belgium; 3 Ministry of Public Health, 9 Rue Printsy Ratsimamanga, Antananarivo 101, Madagascartsivahinypaubert@yahoo.fr (P.T.)

**Keywords:** vaccination, zero-dose children, qualitative study, Madagascar, perceptions

## Abstract

***Background/Objectives*:** Low vaccination coverage and the persistence of zero-dose children remain the principal challenges for immunization efforts in Madagascar. To address these barriers, a socio-anthropological study was conducted to identify the determinants of both vaccination and non-vaccination in eight districts of the country. ***Methods***: District selection was based primarily on immunization performance—specifically the proportion of zero-dose children—along with criteria of geographic and cultural diversity. A qualitative approach was employed, comprising 162 semi-structured individual interviews and 41 focus group discussions with key informants, including political–administrative, religious, and traditional authorities, healthcare workers, community health workers, and parents. ***Results***: Overall, the benefits of vaccination were widely acknowledged by the population. Anti-vaccine rumors were found to be sporadic and, due to their provisional nature, potentially reversible even among those who relay them. Beyond conventional barriers such as scheduling constraints and limited accessibility, fluctuating motivation among community health workers and structural challenges affecting their work emerged as notable findings. Conversely, factors promoting vaccine acceptance were associated with trust in the vaccinators themselves and with a good understanding of vaccination-related issues, fostered through increased and context-specific sensitization efforts. ***Conclusions***: In conclusion, no evidence was found to associate contexts such as rural settings or low-performing vaccination areas with lower vaccine acceptance. Similarly, anti-vaccine rumors were not confined to any particular category or group. Ultimately, the main obstacles are the prioritization of economic risk and concerns about potential side effects. The primary recommendation concerns strengthening awareness-raising efforts, while strengthening trust and improving the working conditions of community health workers.

## 1. Introduction

In 2024, despite joint efforts and initiatives such as the Big Catch-Up launched in April 2023 by Gavi, the Vaccine Alliance, and other partners (WHO, UNICEF) to increase the world immunization rate, an estimated 14.3 million children worldwide remained zero-dose, and 5.6 million were under-vaccinated [1]. Low-income countries are disproportionately affected by these shortcomings [1]. Notably, “half of the world’s 20 countries with the highest number of zero-dose children are located in Africa” [2].

Madagascar is no exception. A decline in immunization performance has been observed since 2020, when Penta3 coverage stood at 86% [3]. Following the COVID-19 pandemic, coverage dropped to 68% in 2022 [3]. A modest recovery was observed in August 2024 and 2025, reaching 81% [3]. The national target, however, is to achieve 95% coverage for all antigens.

The situation is particularly concerning with respect to the persistence of so-called “zero-dose” children. As of July 2024, nearly half (48%) of the country’s health districts reported that zero-dose children represented more than 10% of the target population for routine vaccines [4]. In some localities, this proportion reached 38%, despite the implementation of Periodic Intensification of Routine Immunization (PIRI), specifically designed to reach zero-dose and under-vaccinated children [4].

Several studies have sought to identify the main barriers to vaccination in Madagascar. The Lot Quality Assurance Sampling (LQAS) survey conducted after the first round of the 2024 Supplementary Immunization Activities (SIA) for polio elimination revealed that the leading reason for non-vaccination was child absence (57%) [5]. In addition, refusals and alternative reasons accounted for 17% and 6%, respectively [5]. Although often underestimated in quantitative studies, beliefs—both individual and community-level—ranked first among these alternative reasons [5], warranting particular attention.

A number of investigations have already explored these issues. A socio-anthropological study conducted in 2018 in the Menabe region (comprising five districts) in southwestern Madagascar documented maternal reluctance toward vaccination campaigns, despite positive attitudes toward routine immunization. These campaigns were perceived as excessive, repetitive, and driven by political interests, resulting in significant mistrust toward vaccination agents [6].

Similarly, another study, conducted in 2006, highlighted the central role of trust—particularly trust in healthcare workers—in fostering vaccine acceptance [7]. The opinion of influential community members was also considered critically important [7].

Building on this body of evidence, the present study aims to fill remaining gaps concerning the prioritization of vaccination, the typology of circulating rumors, and prevailing perceptions of vaccines. The primary objective is to identify community perceptions and attitudes toward vaccination in Madagascar, while taking into account structural barriers affecting immunization performance.

## 2. Materials and Methods

**Figure 1:**
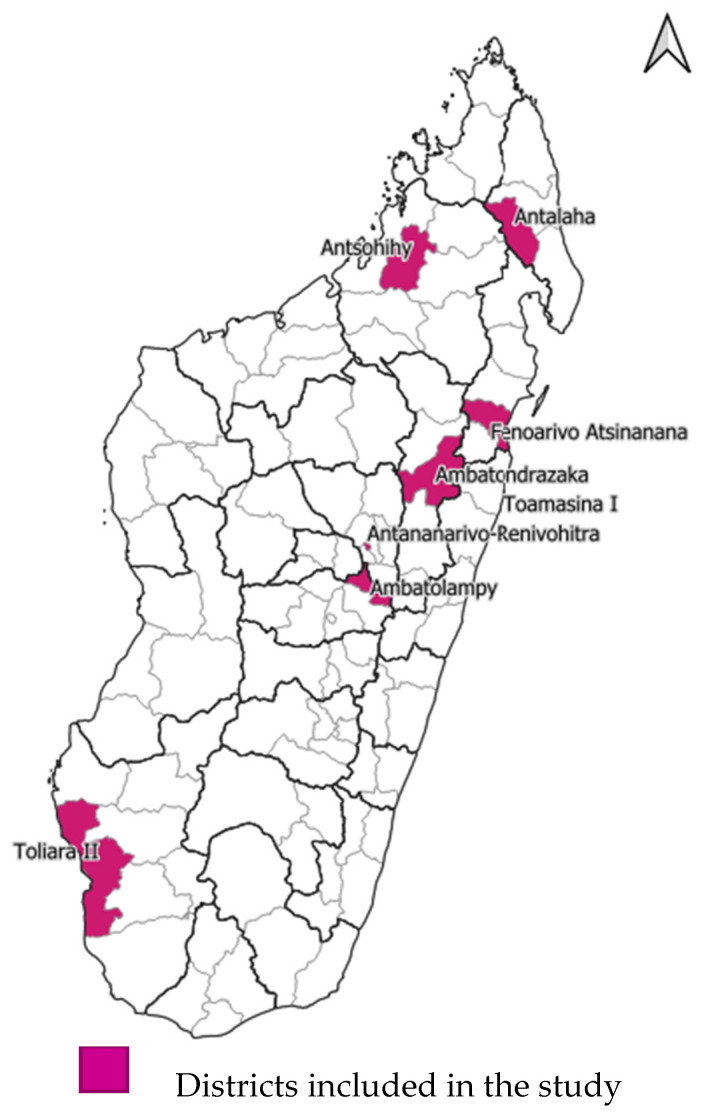
Map of the districts covered by the study.

The study was conducted across eight of Madagascar’s 114 health districts. The districts concerned are indicated in Figure 1: selection prioritized districts with both high and low proportions of zero-dose children.

In addition, the selected districts are located within geographic areas that broadly reflect cultural specificities. In Madagascar, ethnic groups are generally distributed across distinct territorial zones (see Supplementary File S1 for details). The eight districts included in the study were, therefore, Antalaha (North), Antsohihy (West), Toliara II (South), Fenoarivo Atsinanana (East), Ambatolampy (Central Highlands), and the following urban agglomerations: Antananarivo-Renivohitra, Ambatondrazaka, and Toamasina I.

This study employed an interpretive descriptive design, a qualitative research approach that integrates phenomenological and ethnographic perspectives [8]. In other words, it examines participants’ lived experiences and representations while also attending to their broader cultural systems. Ethical approval was obtained from the Ethical Review Committee for Biomedical Research of the Institut National de Santé Publique et Communautaire (INSPC), Mahamasina, Antananarivo (Approval Code: N° 019/2024—CEERINSPC/IRB; 30 December 2024). All participants provided verbal consent, recorded at the start of each interview. Verbal consent was used due to the Malagasy context, characterized by an oral culture, where written consent may raise suspicion and limit participation.

The study spanned approximately seven months, with data collection conducted from 6 to 10 January 2025.

The study population consisted of the following key informants:

- Opinion leaders, including political–administrative, religious, and traditional authorities as well as traditional birth attendants and traditional healers (in French APART: Autorités Politico-Administratives, Religieuses et Traditionnelles), selected for their high level of influence within their communities. This category (APART) is widely found in official public health documents in Madagascar and seeks to map the most influential figures at the community level; see, for instance, [9,10].
- Healthcare workers (based in primary health centers) and community health workers (or CHWs, operating at the community level), selected due to their central and well-recognized roles in vaccination activities.
- Mothers of children under five years of age, both pro-vaccination and vaccine-hesitant, who are at the core of household vaccination decision-making.

These categories were targeted for semi-structured individual interviews due to the specificity of their profiles and the need to capture nuanced and potentially sensitive perspectives.

In total, 162 semi-structured individual interviews were conducted during the data collection phase (see Supplementary File S2 for details).

In addition, fathers and grandfathers—as community elders—participated in focus group discussions (FGDs) to explore key issues related to child vaccination. The selection of this second category was informed by the patriarchal and gerontocratic nature of Malagasy society [11], an aspect considered more likely to emerge in group settings due to its deep social embeddedness.

A total of 41 FGDs were conducted (see Supplementary File S2 for details).

Altogether, 162 semi-structured interviews and 41 FGDs were carried out across the selected study sites. Saturation was reached for each participant subgroup.

Both individual and group interviews explored knowledge, representations, and community attitudes toward vaccination, including cross-cutting themes such as perceptions of vaccines, circulating rumors, and presumed barriers to immunization.

For each district, a team of two field investigators supervised by one coordinator was deployed. Within each district, the selection of communes was guided by the District Management Teams (in French: Équipe de Management de District or EMAD), based on the previously defined criteria, particularly the vaccination performance of primary health centers. Thus, primary healthcare centers with the lowest performance in low-vaccination districts, as well as those with the highest performance in high-vaccination districts, were selected. Upon arrival in the selected communes, data collectors were supported by local guides. Participant recruitment followed a convenience sampling approach. Semi-structured interviews and FGDs were audio-recorded after verbal consent was obtained, following assurances of complete anonymity in data processing and dissemination.

Transcription and translation began immediately after data collection ended (15 January 2025) and were completed during the first week of February 2025.

Subsequently, coding was undertaken using NVivo software (version 12). At this stage, a separate team of three researchers took over the analytical process.

A sequential thematic analysis was conducted. To initiate the coding process, a subset of interviews was randomly selected to generate preliminary codes that informed subsequent stages of data analysis.

Furthermore, given that qualitative studies typically rely on an iterative approach, the research team conducted daily debriefing sessions during the empirical data collection phase. These sessions allowed for adjustments to the interview guides, facilitated triangulation, and helped correct deviations from the original intent of the study.

## 3. Results

Overall, the findings highlighted community perceptions of vaccination, concerns regarding adverse events, circulating anti-vaccination representations, the respective stances of religious and traditional leaders, parental hesitancy, and the predominance of economic concerns. Structural barriers—such as issues related to payment of community health agents, challenges in accessing vaccination sites, and constraints linked to vaccination schedules—also emerged. Each of these themes is detailed below.

### 3.1. Widespread Recognition of the Importance of Vaccination Despite Persistent Misconceptions

The results reveal unanimous acknowledgment of the importance of vaccination among parents as well as political–administrative, religious, and traditional authorities. Although detailed knowledge of specific vaccines (e.g., antigen names and associated target diseases) was often limited, all groups emphasized the public utility of immunization. Even traditional healers and religious leaders—frequently associated with vaccine hesitancy—expressed general support for vaccination.

“Immunity,” “protection”: these two terms were consistently repeated across participant categories, reflecting a solid baseline of community-level vaccine literacy.

However, certain misconceptions and potentially harmful misunderstandings persist.

A first misconception concerns the perception of vaccines as a panacea, exemplified by the statement of a birth attendant in Antananarivo-Renivohitra: “Vaccines protect against absolutely all diseases.”

This belief suggests an assumption that vaccination provides universal protection irrespective of pathogen or disease.

Another recurring misunderstanding is the conflation of vaccines with injections more broadly—namely, the idea that any injectable treatment is a vaccine.

When asked about a significant vaccination-related anecdote involving himself and/or his relatives, an administrative official in Antsohihy responded by referring to “injectable treatments” that reportedly cured him of a high fever, an experience he indicated had also been shared by his son.

This account illustrates the confusion between therapeutic injections and preventive vaccination, as the participant associated the former with a vaccination-related anecdote.

A similar perception was reported by a political–administrative, religious, and traditional authority member in Antananarivo-Renivohitra, who stated:

“Nothing can fight malaria as effectively as the vaccine. Oral treatments do not work; only the vaccine does. If we had not taken my [other] son to get injected (*nitsindrona*), he probably would not have survived”.

This excerpt again highlights the confusion between vaccination and injectable treatment. It also introduces another common misconception: the attribution of vaccines to diseases they do not yet prevent, or reference to vaccines not available in Madagascar. The malaria vaccine, for instance, was mentioned repeatedly during fieldwork, as illustrated in the previous excerpt, despite not being available in the country.

Additional misconceptions concern the perceived eligibility for vaccination. An evangelical pastor in Antalaha stated: “I think that vulnerable people should be vaccinated—those who are not in good health. But healthy and strong people do not really need it”.

Similarly, the assertion that vaccination should be limited to children only was also encountered in the study setting.

These misconceptions were reported predominantly among political–administrative, religious, and traditional leaders, as well as traditional birth attendants and traditional healers.

### 3.2. Concerns About Adverse Events and Fear of Injections

Another prominent theme concerns parental fears related to adverse events following immunization, despite frequent reassurance from healthcare workers. A father interviewed in Toamasina I summarized a widely shared concern: “What worries us are the fatigue, fever, and health disturbances that occur after vaccination and last until the body adjusts. Maybe that is why people are less inclined to get vaccinated”.

A fear of injections also emerged as a recurring issue, as illustrated by the words of an administrative official in Antalaha: “In general, there is a problem among parents when the vaccines administered are injectable. Some run away with their children to avoid vaccination”.

The aforementioned fears fuel a wide range of rumors and tactics aimed at avoiding vaccination, including fleeing when vaccinators approach, hiding the child during their visit, and various other strategies.

### 3.3. Scattered Rumors and the Absence of a Unified Anti-Vaccine Movement or Ideology

There is no unified anti-vaccine movement in Madagascar: indeed, neither the literature review nor the field investigations revealed the existence of such movements. Instead, a proliferation of scattered rumors is observed.

In Madagascar, the rumors encountered most frequently accused either the State, foreign powers, or obscure conglomerates perceived as malevolent:

“People say, for example, that vaccines were designed to reduce the number of people on Earth.”

(Interview with a traditional healer, Toamasina I District)

Vaccination campaigns are strongly singled out in this context:

“There is routine vaccination, but there are also mass vaccination campaigns, and whenever these occur, people are on their guard: they think it is a pretext to introduce new diseases. They say there are too many people in Madagascar and that this is how the excess will be eliminated”.

(Interview with a traditional healer, Fenoarivo Atsinanana District)

COVID-19 vaccines were widely associated with such troubling intentions:

“During the Covid pandemic, rumors were widespread: ‘This is something invented; it is not a natural disease. They want to reduce the population because there are too many of us. This vaccine causes sterility and shortens life expectancy”.

(Interview with a traditional chief, Toamasina I District)

However, these hostile opinions do not appear to be deeply entrenched. With sustained community sensitization, health personnel may be able to reverse them, as illustrated in the following case:

“[…] During a polio vaccination campaign, the rumor that the vaccine spread diseases intensified, and we therefore conducted a field visit to meet a man and a woman who were relaying these theories. After asking them to elaborate on their views, the man suddenly became agitated (…) He claimed that we were spreading harmful germs through the vaccine, hence his aggressive behaviour. We reassured him by explaining that this was a presidential initiative and that it was highly unlikely that the president would want to exterminate us all; he would rather aim to improve public health indicators, we reiterated. Eventually, the man ended up being convinced”.

(Interview with a health worker, Ambatondrazaka District)

### 3.4. Ambiguous Stance of Certain Religious Movements and the Repeated Support of Traditional Authorities and Traditional Healers for Vaccination

A recurrent theme emerged throughout the investigation: followers of new religious groups were reportedly the most resistant to this type of public health intervention:

“It is mainly the new religious groups—if not outright sects—that are hostile to vaccination: their members often invoke the pastor’s prohibition in this regard”

(Interview with a traditional healer, Antananarivo-Renivohitra District)

The main groups singled out were affiliated with evangelical movements, a prolific branch of neo-Protestantism.

Their pastors were therefore approached but immediately dismissed any allegation that their followers were formally forbidden from being vaccinated. According to them, the decision to vaccinate rests entirely with the individual. As such, no sensitization or encouragement toward vaccination could be delivered to the congregation.

However, where the stance of these religious leaders appears most ambiguous is in the attitudes they personally model—attitudes marked by more or less ostentatious defiance toward vaccination, which may inspire imitation among their followers:

“Personally, I believe that divine protection is sufficient and that vaccines are therefore unnecessary. I have been sick before and recovered without medical treatment, so I am convinced that my faith protects me”.

(Interview with a pastor from an evangelical church)

“As for me, I have four children, but only the first was vaccinated, back when I had not yet converted. Once I truly discovered the faith, I realized that the blood of Christ is the only real vaccine and the ultimate remedy”.

(Interview with a pastor from another evangelical movement)

In stark contrast to these religious authorities, traditional authorities such as traditional healers emerged as unexpectedly strong allies of vaccination.

It is worth noting that all traditional healers consulted emphasized the complementarity between traditional medicine and Western medicine. A traditional birth attendant working in Antalaha District summarized this position unequivocally, stating that “[traditional medicine and Western medicine] are each worth their weight in gold,” with traditional medicine often addressing presumed cases of sorcery, while Western medicine handles conditions classified within biomedical science.

In the same vein, all healers and traditional birth attendants approached—even those most steeped in mysticism—consistently reported promoting vaccination and considering it a priority, even a “fundamental imperative” (Interview with a traditional birth attendant, Fenoarivo Atsinanana District).

### 3.5. Parental Hesitancy Toward Vaccination in Schools

The contribution of private schools to achieving national vaccination targets appears uncertain.

Indeed, a faith-based school in the Ambatondrazaka District faced protests from several parents after scheduling on-site vaccination sessions for its pupils. This incident led to the cancellation of such activities in the future, a decision that subsequently influenced other private schools of the same type.

### 3.6. Prioritization of Economic Concerns over Health Concerns

Overall, the investigations revealed that economic risk outweighs health risk in the collective mindset; people are primarily preoccupied with economic survival.

Most participants attested to this hierarchy of concerns, as illustrated by the following account:

“If you take the time to really observe people in their daily lives, it becomes clear that health ranks second after the imperative of having something to eat. Lack of means is their main concern; daily sustenance is the priority. Health comes only afterwards… In fact, people are mainly taken up by their work activities, which is why health workers conducting community outreach often find only two or three households available during their rounds”.

(Interview with a father, Antsohihy District)

That said, a closer examination shows that people do not disregard health concerns. Lacking financial means and medical literacy, they tend instead to rely on alternative and traditional medicine, all within a worldview that sacralizes life:

“Yes, people are very careful (smiles), because they fear for their lives (smiles). They come to see me for treatment, and that already shows they take their health seriously. They are afraid of dying, so whether through traditional or modern medicine, they always look for a way to heal themselves”.

(Interview with a traditional healer, Fenoarivo Atsinanana District)

### 3.7. A Major Structural Barrier to Vaccination: Fluctuating Motivation Among Community Health Workers and Their Limited Influence in Urban Areas

The focus on representations also sheds light on structural barriers hampering the success of vaccination activities. Low motivation among community health workers (CHWs), stemming from absent or insufficient compensation, emerged as a recurrent theme:

“Some CHWs are not interested in what they are assigned unless there is some [financial] motivation behind it”.

(Interview with a health worker, Antananarivo-Renivohitra District).

A clear imbalance between workload and remuneration was cited as the obvious source of fluctuating engagement levels.

This fluctuation would be amplified by the lack of recognition and by the condescending attitudes of urban populations toward community health workers:

“I admit that community health workers know their field well, but I don’t know… It seems to me that the community pays more attention when other actors intervene”.

(Interview with a health worker, Antananarivo-Renivohitra District).

The same health worker elaborated, proposing a hypothesis:

“The problem is that [community health workers] are people from the community, and that may be why they are not accepted. We see them every day. I don’t know if you understand: there is a kind of condescension, an underestimation of these workers!”

In this context, it is important to note that community health workers do not wear uniforms and only display a simple badge. The contrast is striking when compared to a professional health worker; only well-informed community members are likely to recognize them. An active CHW in Antsohihy District strongly emphasized this point: “Wearing distinctive clothing would be very helpful; it would make a big difference”.

Regarding other obstacles, most CHWs approached were somewhat reticent, focusing solely on technical aspects despite the investigators’ insistence:

“The main difficulty is sensitizing parents, because they are often suspicious, or the wife invokes her husband’s categorical objection”.

(Interview with a CHW, Antananarivo-Renivohitra District)

However, some CHWs openly raised the same issue previously highlighted by health workers. A CHW working in Antsohihy District openly stated:

“We do not receive a salary. Therefore, we request more compensation because life is difficult while we must provide for our families. That is why we ask for support”.

Variations of these problems were also reported, including delays in the disbursement of allowances that were already considered insufficient.

### 3.8. Other Structural Barriers: Time and Distance Constraints

In addition to the predominant obstacle of under-compensation for CHWs, other structural barriers emerged during the investigation.

The issue of accessibility to vaccination centers was repeatedly highlighted, particularly in rural areas:

“Another barrier [to vaccination] that I haven’t mentioned yet is remoteness. In rural areas, the roads are poor and impassable by motorcycle. If they were at least passable by motorcycle, that would be better. But in some remote regions, such as Analanjirofo, where one cannot even access by motorcycle, this really prevents the majority of people from taking their children to be vaccinated”.

(Interview with a traditional healer, Fenoarivo Atsinanana District)

A politico-administrative figure from Antalaha District further linked remoteness, isolation, and lack of infrastructure in a candid assessment: “The lack of infrastructure is another salient barrier, related to the remoteness of those already available. In such cases, people are instantly discouraged at the thought of having to travel kilometers to take their children for vaccination”.

In a way, these issues intertwine distance- and time-related constraints, one being inseparable from the other. However, the time-related constraint also reflects the prioritization of economic and structural concerns, as noted previously.

### 3.9. Trust and Vaccine Literacy: Two Pillars of Uptake

While a large portion of the results focuses on barriers to vaccination, their converse was also explored during the study. Countering the narratives of fragmented trust, particularly toward health authorities, participants highlighted the critical role of trust in health workers and community health workers (CHWs) in vaccine uptake:

“There are those who say that if it is them [the CHWs and health workers familiar to them] conducting the awareness, then this [the vaccine] can only be in favor of something safe. In such cases, people accept”.

(Interview with a traditional healer, Toamasina I District).

This trust is closely linked to effective sensitization, according to respondents:

“Sensitization—that is the key,” stated an administrative official based in Fenoarivo Atsinanana, continuing by also invoking, in the same vein, “education.”

In this perspective, another administrative official, based in Ambatondrazaka, emphasized persuasion through highlighting the harmful effects of non-vaccination:

“Persuasion is very simple: it is extremely difficult to find money nowadays, especially for healthcare, because medicine prices are simply exorbitant. It is inconceivably costly, so to prevent serious illness, we simply urge residents to bring their children for vaccination”.

(Interview with an administrative official, Ambatondrazaka District)

Other participants suggested similar acceptance factors, rooted in an existential fear of disease and mortality.

## 4. Discussion and Recommendations

### 4.1. Discussion

#### 4.1.1. Misconceptions About Vaccination and Threats to Herd Immunity

Several misconceptions regarding vaccines and vaccination more broadly were identified in the results. In isolation, these beliefs might have relatively minor consequences; however, they pose risks to the proper functioning of institutional vaccination activities.

For instance, the belief in a “panacea vaccine” may concomitantly generate disproportionate expectations regarding vaccines. Such expectations can subsequently lead to disappointment and frustration in the event of illness, ultimately fostering narratives about the presumed inefficacy of vaccines [12].

Similarly, the conflation of all injections with vaccines follows the same logic [12], potentially giving rise to inappropriate expectations regarding both vaccine and non-vaccine injections, thereby posing the same risk of discourses questioning vaccine effectiveness. Misunderstandings regarding the actual diseases prevented by vaccines may also trigger a comparable detrimental feedback loop.

Finally, misconceptions concerning target populations can adversely affect vaccination activities by generating disinterest among groups that perceive themselves as not concerned—for example, adults.

In general, the ideas of a “panacea vaccine,” the interchangeability of injections, and vaccines as protection against an excessive number of diseases reflect a certain discursive exaggeration [12]. As noted above, these conflations were particularly observed among the group comprising opinion leaders: politico-administrative, religious, and traditional authorities, as well as traditional birth attendants and traditional healers.

In this context, potential response biases may have influenced the findings, considering that data collectors were operating under the auspices of the World Health Organization (WHO), a longstanding governmental partner. This aspect will be discussed in greater detail in the section dedicated to the study’s limitations.

#### 4.1.2. Philosophies and “Ready-Made” Anti-Vaccination Thinking

As noted, no unified anti-vaccination movement exists in Madagascar; rather, hostile rumors about vaccination are scattered, modulated, and refracted according to individual actors.

They align, however, with the typology of anti-vaccination philosophies proposed by Olivier Jourdain, which identifies neo-Malthusianism, naturalism, libertarianism, and various esoteric currents such as anthroposophy [13]. The first category resonates with participants’ statements denouncing a conspiracy centered on population reduction to serve obscure ends.

Nevertheless, none of these philosophies has so far given rise to organized movements or any federating momentum; their function being seemingly limited to enriching the individual’s knowledge base or, in other words, serving as provisional opinions, “ready-made” ways of thinking.

Their proliferation can be partially attributed to eroding trust in public authorities. As Jourdain notes: “Trust in vaccination is closely correlated with the population’s trust in its political and scientific institutions” [13]. Indeed, as observed, the state has been at the center of numerous rumors hostile to vaccination, alongside healthcare workers who are unknown to the community.

Moreover, identifying a typical “anti-vaxxer” profile proved difficult, as anticipated by Jourdain [13]. The reason is straightforward: anti-vaccination opinions are not the prerogative of any particular social class or category. They are highly heterogeneous, drawing on foundations as diverse as esotericism or grievances against the government [13].

The sociological mechanisms underlying rumor diffusion merit some attention to better understand the nature of these rumors. The scientific literature on the subject is abundant and has undergone several evolutions [14].

For public health studies, rumors as mostly seen are the contextualized expression of a “breakdown in trust” rather than as the product of “pharmacological objections” [15]. Anthropologist Heidi Larson illustrates this with the anti-vaccine wave in the Middle East, which she attributes more to anti-American sentiment and geopolitical representations than to medical facts [15].

In this sense, real or perceived power relations play a predominant role, leaving a lasting imprint on collective opinion. Indeed, the reversibility of certain anti-vaccine opinions, as we saw in the results, may be explained as a form of resistance against institutional power rather than as the result of strictly medical skepticism.

#### 4.1.3. Injection Phobia, Biopolitics, and Risk Culture

The injection phobia reported by participants did not stem from cultural prohibitions but rather reflected concerns about potential side effects.

This phenomenon can be understood as a protective response to biopower as exercised through national vaccination policies. Michel Foucault’s concept of biopower describes the exercise of power over life and bodies, forming the basis of “biopolitics,” a mode of governance that regulates populations [16].

Applied to vaccination, this framework highlights the interplay between individual perceptions, public health interventions, and societal regulation. This extract summarizes this tension, stating: “The vaccine raises concerns that are both personal and societal: it intersects with the biological and political, identity and social norms, disease and cure, individual freedom and collective responsibility, life with pathogens and their elimination” [17].

Originating in the context of the rationalization of Western societies, biopower also tends to impose a rationalist diktat that resonates with the notion of a “risk culture,” itself defined as a rationalist perception of risks, particularly health-related [18].

It follows that, in a context marked by a crisis of trust toward public authorities, these forms of biopolitics are received with suspicion or even hostility by a significant portion of the population, manifesting as opposition to vaccination. This is particularly noticeable, as we have observed, during vaccination campaigns where the governmental narrative and the repetitive nature of these campaigns generate mistrust.

Furthermore, the use of traditional medicine and economic imperatives also suggests a different hierarchy of risks, outside the framework of classical risk culture. In this context, concerns related to economic risks prevail, but they are not really opposed to those pertaining to health. A culture of health-related risk does indeed exist, yet it does not necessarily rely on the Western rationalist tradition.

#### 4.1.4. Prohibition of Vaccination in Certain Faith-Based Schools: A Significant Barrier

Some faith-based schools have actively prohibited vaccination campaigns to prevent potential parental protests, creating a substantial barrier to reaching vaccination targets.

Generally, this stance does not reflect outright opposition to vaccination but rather a logic of adaptation and compromise, linked to the entrepreneurial dimension of the school. The term “school market” has been used to capture this aspect of client retention specific to the educational establishment [19]. Indeed, school administrations do not take explicit positions on vaccination itself. Instead, they invoke the fundamentally individual nature of vaccination decisions, thereby responding appropriately to parental expectations—the central actors within the school system.

In this sense, the schools’ strategic approach exploits a paradox inherent in any vaccination policy: the constant oscillation between coercive intent and benevolent encouragement. This paradox reflects a broader paradigm, that of health democracy, a branch of the global democratic model. Adopting such a paradigm inherently entails negotiating this kind of paradox [20].

Nevertheless, school participation remains critical to the success of vaccination campaigns. For instance, the introduction of the HPV vaccine in Madagascar, during the pilot phase from 2013 to 2015, highlighted this point, demonstrating the centrality of schools within the vaccination strategy [21].

#### 4.1.5. Social Stratification and Multidimensional Marginalization of Community Health Workers

As previously noted, community health workers (CHWs) frequently report financial precarity, citing both inadequate and delayed compensation. This reflects a form of social stratification that renders their occupational situation precarious.

However, this stratification is not limited to financial aspects. In urban settings, CHWs are additionally subjected to community condescension. The symbolic capital that confers desirability to the role in rural contexts is notably absent in urban environments [22].

At this level, a multidimensional form of domination emerges, widening the gap between CHWs and other health personnel. The semi-formal status of CHWs becomes a central point of discussion, particularly within the hierarchical structure of the ostensibly community-based health system.

This challenge resonates with global patterns, as highlighted by the Community Health Impact Coalition:

“Half of all CHWs in low- and middle-income countries (LMICs) are unpaid, including 86% in Africa” [23].

“CHWs represent 14% of the total health workforce in Africa, yet only 14% of them are salaried” [23].

### 4.2. Recommendations

#### 4.2.1. Financial and Symbolic Incentives for Community Health Workers

The current remuneration system for CHWs in Madagascar still largely aligns them with volunteer status, representing a major structural barrier despite ongoing efforts. A performance-based model was piloted during the Periodic Intensification of Routine Immunization (PIRI) from 2 to 6 June 2025, in addition to the regular allowances received by CHWs. The results were encouraging: these intensive vaccination activities reached an unprecedented peak in immunizing children aged 12–59 months (120,000 children vaccinated compared with approximately 20,000 during previous PIRIs), a success largely attributed to the CHWs’ financial incentives [24].

Overall, this incentive system proved effective, although more time is needed to fully evaluate its cost–benefit ratio. Nonetheless, it remains in its early stages, and systematic implementation remains a challenge. The volunteer model still predominates across the board. The WHO has issued guidance [25] suggesting, without explicitly mandating base remuneration, that CHWs should be “compensated through a financial gratification system commensurate with the requirements and complexity of their work, the number of hours worked, their training, and the roles they assume” (Free translation) [25].

Other countries provide instructive examples. In Brazil, CHWs are formally recognized as state employees with a 40-h workweek and a minimum salary [26]. Annual updates to remuneration, along with risk-based bonuses for hazardous working conditions, further strengthen the program [26]. These measures have contributed to outstanding outcomes: “[Brazil’s community health program] helped the country reduce under-five mortality by 75%, maternal mortality by nearly 60%, and doubled immunization coverage, achieving near-universal immunization” [27].

In Nigeria, CHWs occupy a pivotal role despite not being formally integrated into the civil service. A minimum salary is provided, typically funded by technical and financial partners. Notably, CHWs in this context often possess high-level qualifications [28]. Drawing from these international examples, we recommend that Madagascar adopt an equitable distribution of responsibilities among technical and financial partners to ensure fair remuneration. Central-level coordination will be essential, ideally culminating in the establishment of a minimum salary for CHWs.

Beyond financial incentives, as indicated by both health staff and CHWs themselves, a lack of symbolic capital—prestige and visibility—also constrains CHWs, particularly in urban areas [29]. Addressing this issue could begin with formalizing CHWs’ status or at least establishing a baseline remuneration. Additionally, organizing community meetings for the official presentation of CHWs—aligned with relevant local occasions—could further enhance their symbolic status and recognition within the community.

In urban areas, those meetings can also be facilitated through appropriate media outlets.

Along the same lines, standardization of the community register used by CHWs constitutes another priority. This standardization should be accompanied by training on proper completion procedures. Effective monitoring mechanisms complete this chain of interventions.

#### 4.2.2. Engagement of Religious Leaders in Vaccination

As noted, some religious movements, particularly certain evangelical currents, have proven to be “false friends” regarding vaccination. In these cases, sustained advocacy is necessary, highlighting the support provided by other religious groups as a point of comparison.

For instance, a representative of the Christian Women’s Association “Dorkasy” encouraged women to receive the COVID-19 vaccine [30] in the aftermath of the pandemic. A pastor from the FJKM church in Antananarivo joined her in relaying this message [30]. Similarly, the Lutheran Church in southern Madagascar actively promoted vaccination within local communities [31].

Advocacy targeting ambiguous or resistant religious communities can build on these examples in addition to presenting factual evidence on vaccination. Actors across multiple levels can participate: relevant ministries such as the Ministry of Public Health, the Ministry of Communication and Culture, and the Ministry of Population and Social Affairs; religious leaders supportive of vaccination from other faiths; and interfaith organizations that can serve as bridges and dialogue spaces, such as the Confederation of Churches and Evangelical Associations of Madagascar (in French: *Confédération des Églises et Associations Évangéliques de Madagascar* or CEAEM).

Moreover, technical and financial partners such as the WHO and UNICEF can play a key role as mediators or by creating new spaces for dialogue.

#### 4.2.3. Engagement of Schools in Vaccination

Complementing the engagement of religious organizations, advocacy can also target resistant schools by referencing the experiences of those institutions that support vaccination activities, while continuously promoting dialogue with parents.

Upstream, integrating educational sessions for students on vaccination-related topics may represent a promising avenue. The objective is to foster early literacy on vaccination to prepare parents for the possibility of vaccination activities within the school. For illustration, such sessions are among the practices favored by the French government to improve national vaccination coverage, from primary school [32] through secondary school, with guides provided for teachers supportive of the initiative [33].

These initiatives fall under the joint responsibility of the Ministry of National Education and the Ministry of Public Health at the institutional level, with the support of technical and financial partners such as WHO, UNICEF, and UNESCO. At another level, education professionals, renowned intellectuals, and pro-vaccination parents can also contribute to these efforts.

#### 4.2.4. Strengthening Mobile and Outreach Strategies and Adapting Vaccination Schedules

Given the persistent challenge of limited access to vaccination centers, it is evident that strengthening both outreach and mobile vaccination strategies constitutes a critical component of the overall vaccination effort.

An evaluation of the outreach strategy is underway in Madagascar, following Periodic Intensification of Routine Immunization (PIRI) activities, during which a real-time geospatial tracking system was deployed to monitor vaccination teams.

This system, the Geospatial Tracking System (GTS), has already proven effective in polio eradication campaigns in several African countries, notably in the Republic of Congo during the 9–11 June 2023, anti-polio campaign [34]. In Madagascar, 1906 primary health centers (PHCs) across 66 districts [35] utilized the system during the PIRI from 2 to 6 June 2025. Of these, 1391 teams (73%) submitted at least one report, representing a promising start for the implementation of this tool [35].

The use of this innovation is expected to enable a comprehensive evaluation of the outreach strategy, allowing for adjustments and improvements to address identified gaps.

Additionally, adapting vaccination time slots to align with population needs represents a major avenue for addressing the recurring issue of availability. A study conducted between January and September 2025 in Madagascar by Institut National de Santé Publique et Communautaire (INSPC) examined the feasibility of adjusting vaccination schedules in the districts of Mahanoro and Toamasina I (one of the districts included in the present study) on the east coast of the island [36]. The main findings support the adaptation of vaccination time slots, although this requires consideration of multiple parameters.

The comparison between the two target districts highlighted that while current vaccination time slots are generally acceptable in both urban and rural settings, they remain constrained by professional commitments and daily activities [36]. Regarding the feasibility of potential adjustments, the urban context appears more conducive despite a heavy workload and critical staff rotation [36]. Motivation for such adjustments is high in both contexts, although proximity to vaccination centers is prioritized in urban areas over time slot adjustments [36]. In rural settings, other factors—such as trust in CHWs and adherence to local routines—play a critical role [36].

These findings underscore the importance of contextualizing vaccination schedules, particularly considering the contrasts between rural and urban environments, to better meet population needs.

#### 4.2.5. Additional Recommendations

Further recommendations include the systematic use of beneficiary testimonials, gamification of educational materials, and training of members of local health coordination structures (Health Committees or Co-San, Communal Health Development Committees or CCDS, and Local Consultation Structures or SLC). These measures aim, respectively, to alleviate community fears, counteract habituation to routine sensitization, and address misconceptions.

The idea of gamifying educational content was highlighted by an administrative official in the district of Fenoarivo Atsinanana, citing the sensitization activities carried out by PSI [Population Services International], which travels to remote villages with loudspeakers that disseminate music to attract people. Animated film screenings were also scheduled and reportedly received enthusiastic responses from local populations.

This suggestion, linked to gamification, however, is somewhat counterbalanced by straightforward reminders of the economic consequences of non-vaccination, as well as by the activation of existential fears related to disease and isolation—basic yet highly effective levers, as indicated by participants interviewed earlier.

Study Limitations

First, the study’s duration may represent a significant limitation. Data collection was confined to ten days, rendering it a very short-term snapshot that did not allow for capturing the evolving dynamics of perceptions and behaviors regarding vaccination over a longer period. Individual attitudes can change rapidly in response to specific events. For example, vaccination campaigns, elections, or political, economic, or health crises (such as epidemics) can substantially influence population perceptions and attitudes. A longitudinal follow-up would therefore have been necessary to capture these variations and provide a more comprehensive view of their evolution over time. Indeed, the stability and continuity of representations over time constitute another crucial parameter to consider.

Second, although the study made efforts to select study areas distributed across Madagascar—including the east, west, north, south, and central regions, as well as several urban centers—limitations remain regarding this coverage. Districts were initially chosen based on geographic location and related cultural specificities and subsequently based on vaccination performance indicators (zero-dose rates). However, the notion of cultural representativeness presupposes homogeneity within each geographic area regarding practices and perceptions, which only holds true to a limited extent. A given cultural identity does not necessarily conform to precise geographic boundaries.

Consequently, despite ambitious territorial coverage, the study cannot claim to encompass the full range of cultural particularities across the country.

Moreover, limitations inherent to convenience sampling for participant selection must also be acknowledged, although such limitations are common to most qualitative research. Social desirability bias, particularly among political, administrative, and traditional authorities, is also a factor, as suggested in the discussion, given the tendency of some participants to exaggerate or overstate certain positions. This bias corresponds to “the respondent’s desire to present themselves in a favorable light” [37]. Indeed, the investigators presented themselves under the banner of the WHO.

From this perspective, some accommodating respondents may have overemphasized the virtues of vaccines to be viewed favorably by WHO representatives, a privileged partner of central authorities. Such behavior can be interpreted as a strategic posture aimed at safeguarding their status and acquiring predominantly symbolic (perceived esteem by WHO investigators) and social capital (the “friendship” of WHO envoys).

This bias may extend beyond the respondent’s individual image, potentially enhancing the perceived reputation of the entire community.

A traditional healer in a rural commune of Fenoarivo Atsinanana expressed this sentiment inquisitively: “Why are you asking us these questions? Is it because we are from a rural area?”

His tone implied that the very conduct of the study suggested a condescension toward rural populations, which he may have been eager to counter by portraying his community in the best possible light.

To reduce the aforementioned type of bias, investigators wore only simple badges during both individual interviews and focus group discussions and placed emphasis on the Ministry of Public Health’s intention to improve healthcare services.

In summary, the main vaccination-related representations across the island have been mapped, with particular emphasis on rumors and the prioritization of different risks (health, economic, social, etc.) as deployed by communities. At this stage, however, it remains essential to explore further avenues, such as examining correlations between adherence to specific rumors and variables like education level and occupation. While this approach may not yield a “profile” of the anti-vaccination conspiracist, it can nonetheless generate valuable insights for combating misinformation and disinformation.

## 5. Conclusions

In conclusion, no evidence was found to associate contexts such as rural settings or low-performing vaccination areas with lower vaccine acceptance. Indeed, the benefits of vaccination are generally acknowledged in Madagascar, including across highly contrasting contexts. In addition, similarly, anti-vaccine rumors are not confined to any particular category or group, nor are they linked to any movement. They rather appear as scattered “ready-made opinions” which can potentially be reversed through targeted sensitization efforts.

Non-vaccination in this context results from a complex interplay of factors. The prioritization of economic risk over perceived health risk, along with fear of side effects, occurs against a broader backdrop of eroded trust in authorities, both health-related and governmental. Conversely, key factors promoting vaccine uptake include trust in healthcare workers and community health agents, as well as adequate knowledge of vaccination benefits and health risks.

Needle phobia, perceived as invasive, remains a persistent concern, although it has no specific cultural grounding.

Given these observations, the primary lever to encourage vaccine acceptance is highlighting the economic consequences of non-vaccination while employing more engaging, user-friendly educational materials. Concurrently, mobilizing influential figures—such as religious leaders and political–administrative authorities—along with the institutions they represent (e.g., churches and schools) is critical to reinforce the credibility and acceptability of vaccination initiatives.

Effective sensitization also depends on strengthening both the financial incentives and symbolic capital of community health agents, through equitable distribution of technical and financial partners and increased visibility, particularly in urban areas. Furthermore, optimizing outreach and mobile vaccination strategies, alongside adapting vaccination schedules to population availability, constitutes an additional, essential measure.

Finally, several questions remain unresolved, particularly regarding how to consolidate initial vaccine acceptance and transform it into sustained, active demand in a context where misconceptions and rumors about vaccination remain widespread. These findings underscore the need for a continuous, integrated approach that combines targeted communication, institutional strengthening, and community engagement to support national immunization coverage.

## Data Availability

During the data collection phase, the principles of “beneficence” and “non-maleficence” were rigorously observed, ensuring that the study contributed to participants’ well-being while respecting their autonomy. This was operationalized through obtaining participants’ free and informed consent (in this case, verbal), granting them full discretion to participate or decline inclusion in the study. Furthermore, the procedures for conducting interviews, along with measures for anonymizing and securing data, helped mitigate any potential risks of harm arising from participation.

## Supplementary Materials

The following supporting information can be downloaded at: https://www.mdpi.com/article/10.3390/vaccines14020191/s1, Supplementary File S1: Selection criteria for the study areas. Supplementary File S2: Sample size and features.

## Abbreviations

The following abbreviations are used in this manuscript:

CHW	Community Health Worker
CCDS	Comité Communal de Développement sanitaire (Communal Health Development Committee)
FGD	Focus Group Discussion
GAVI	Global Alliance for Vaccines and Immunization
GTS	Geospatial Tracking System
LQAS	Lot Quality Insurance Sampling
PHC	Primary Health Center
PIRI	Periodic Intensification of Routine Immunization
PSI	Population Services International
SLC	Structure Locale de Concertation (Local Consultation Structure)
UNICEF	United Nations Children’s Fund
WHO	World Health Organization
