# Supplementary material for: Community Perceptions and Attitudes Toward Vaccination in Madagascar
Source: Vaccines (Basel). 2026 Feb 19;14(2):191. doi: 10.3390/vaccines14020191 (PMC12945292; doi:10.3390/vaccines14020191)
Supplement: Supplementary file 1 [file vaccines-14-00191-s001.zip › SUPPLEMENTARY FILE 1 - SELECTION CRITERIA FOR THE STUDY AREAS.pdf]

SUPPLEMENTARY FILE 1 : SELECTION CRITERIA FOR THE STUDY AREAS

Table 1: List of Districts Selected for the Study Based on Cultural Specificities and Vaccination Performance

Zone	Major ethnic groups	Region/District	Zero-dose children rate (07/2024)	Vaccination performance
CENTRAL	Merina, Betsileo	Vakinankaratra/Ambatolampy	0%	High
WEST	Sakalava	Sofia/Antsohihy	7%	High
NORTH	Antakarana, Tsimihety	SAVA/Antalaha	8%	High
SOUTH	Bara, Antandroy, Antanosy, Mahafaly , Mikea	Atsimo Andrefana/Toliara II	4%	High
EAST	Betsimisaraka, Antambahoaka, Antaimoro, Antaifasy, Antaisaka, Sihanaka, Bezanozano , Zafisoro	Analanjirofo/Fenoarivo Atsinanana	22%	Low
Urban areas		Analamanga/Antananarivo Renivohitra	25%	Low
		Alaotra Mangoro/ Ambatondrazaka	38%	Low
		Atsinanana/Toamasina I	5% (12/2024)	High

Sources: Based on MSANP/DPEV, 2024

Vaccination performance

Zero-dose children rate:

>10%: low performance

<10%: high performance
