# Supplementary material for: Community Perceptions and Attitudes Toward Vaccination in Madagascar
Source: Vaccines (Basel). 2026 Feb 19;14(2):191. doi: 10.3390/vaccines14020191 (PMC12945292; doi:10.3390/vaccines14020191)
Supplement: Supplementary file 1 [file vaccines-14-00191-s001.zip › SUPPLEMENTARY FILE 2 SAMPLE SIZE AND FEATURES.pdf]

SUPPLEMENTARY FILE 1: SAMPLE SIZE AND FEATURES

Table 1: Summary Table of Semi-Structured Interviews

District / Subsample	Antalaha	Toliara II	Fenoarivo Atsinanana	Antsohihy	Ambatolampy	Antananarivo-Renivohitra	Ambatondrazaka	Toamasina I	TOTAL
Political-administrative, religious and traditional authorities, TBAs[1], traditional healers	7	7	9	7	7	8	7	7	59
Health workers	2	2	2	2	2	2	2	2	16
Community health workers	5	5	5	5	5	5	5	5	40
Mothers of children under 5	6	6	6	6	5	6	6	6	47
Total	20	20	22	20	19	21	20	20	162

[1] Traditional birth attendants

Regarding the first category (political-administrative, religious and traditional authorities, traditional birth attendants, and traditional healers), 71% of participants were men and 29% women. Among the 17 traditional birth attendants and traditional healers, 10 were traditional birth attendants. Of these, 40% had only completed primary education. These practitioners were also relatively old, with an average age of 61.75 years.

As for health workers, the subsample consisted of 75% women and 25% men. Among them, 46.6% were midwives.

Next came the community health workers, 93% women (and therefore only 7% men), with an education level mostly reaching high school (46%), followed by lower secondary education (34%).

Turning now to the mothers of children under five included in the study: with an average age of 26.47 years, they mostly worked in trade of various goods (38%), were housewives (28%), or worked in the fields (17%). Catholics represented 61% of this sub-sample, followed by members of the Protestant FJKM Church (15%). 55% had the opportunity to study up to junior secondary school, while 25% had to stop at primary level.

Table 2: Summary Table of Focus Group Discussions

District / Subsample	Antalaha	Toliara II	Fenoarivo Atsinanana	Antsohihy	Ambatolampy	Tana Ville	Ambatondrazaka	Toamasina I	**TOTAL**
Fathers and grandfathers	5	5	5	6	5	5	5	5	**41**

The group of fathers and grandfathers involved in the focus groups had an average age of 43.1 years. Their educational background mostly stopped at secondary level (46%), followed by primary level (38%). The most common occupation was farming (68%). Regarding religion, the most represented denominations were Catholicism (54%), Adventism (14%), and Protestantism (FJKM: 7%).
